# Supplementary material for: Upregulated GATA3/miR205-5p Axis Inhibits MFNG Transcription and Reduces the Malignancy of Triple-Negative Breast Cancer
Source: Cancers (Basel). 2022 Jun 22;14(13):3057. doi: 10.3390/cancers14133057 (PMC9264964; doi:10.3390/cancers14133057)
Supplement: Supplementary file 1 [file cancers-14-03057-s001.zip › cancers-1717587-supplementary.pdf]

Supplementary Materials: Upregulated GATA3/miR-205-5p Axis Inhibits MFNG Transcription and Reduces the Malignancy of Triple-Negative Breast Cancer

Samson Mugisha, Xiaotang Di, Doudou Wen, Yuetao Zhao, Xusheng Wu, Shubing Zhang and Hao Jiang

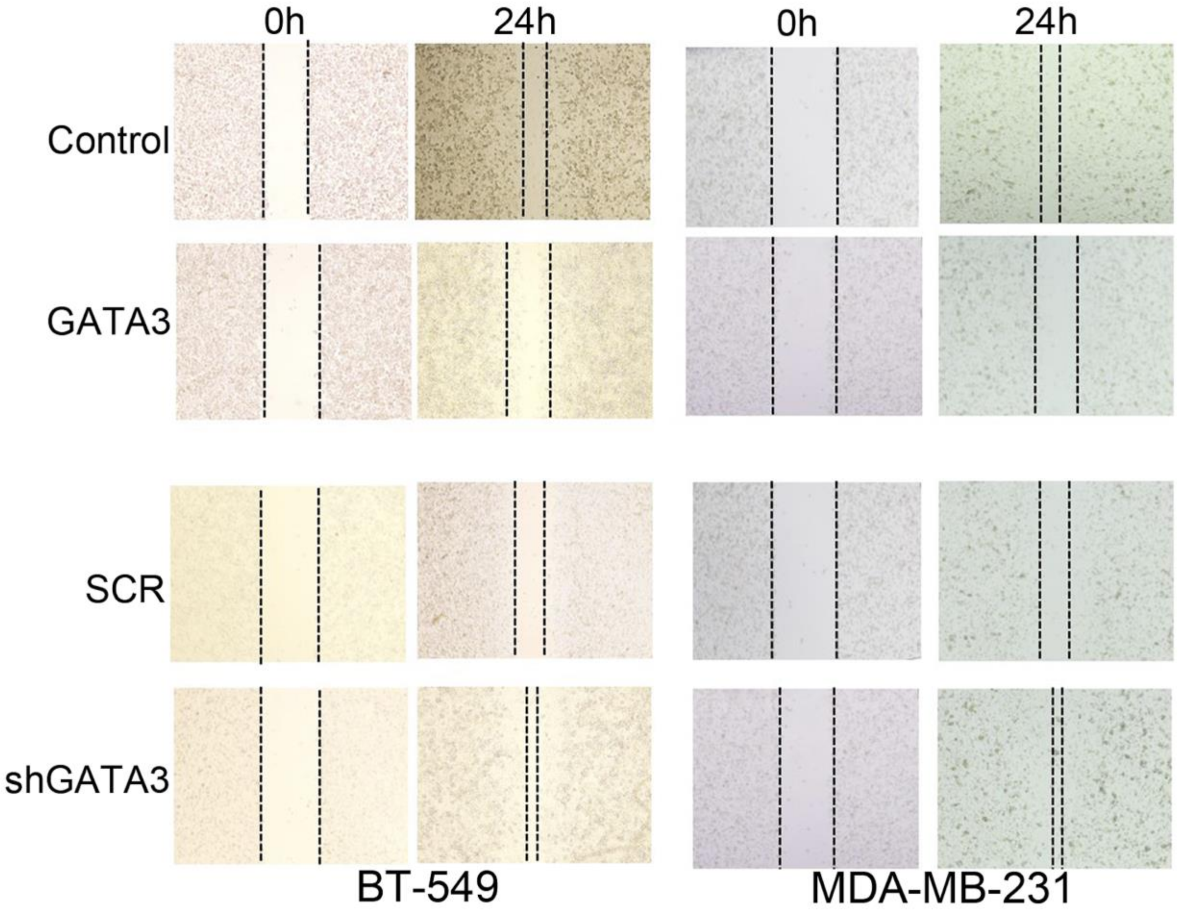

Figure S1. GATA3 suppressed the migration ability of TNBC cells by wound-healing assay. Migration of TNBC cells was observed at 0h and 24h and SCR was short for Scramble.

Figure 2B

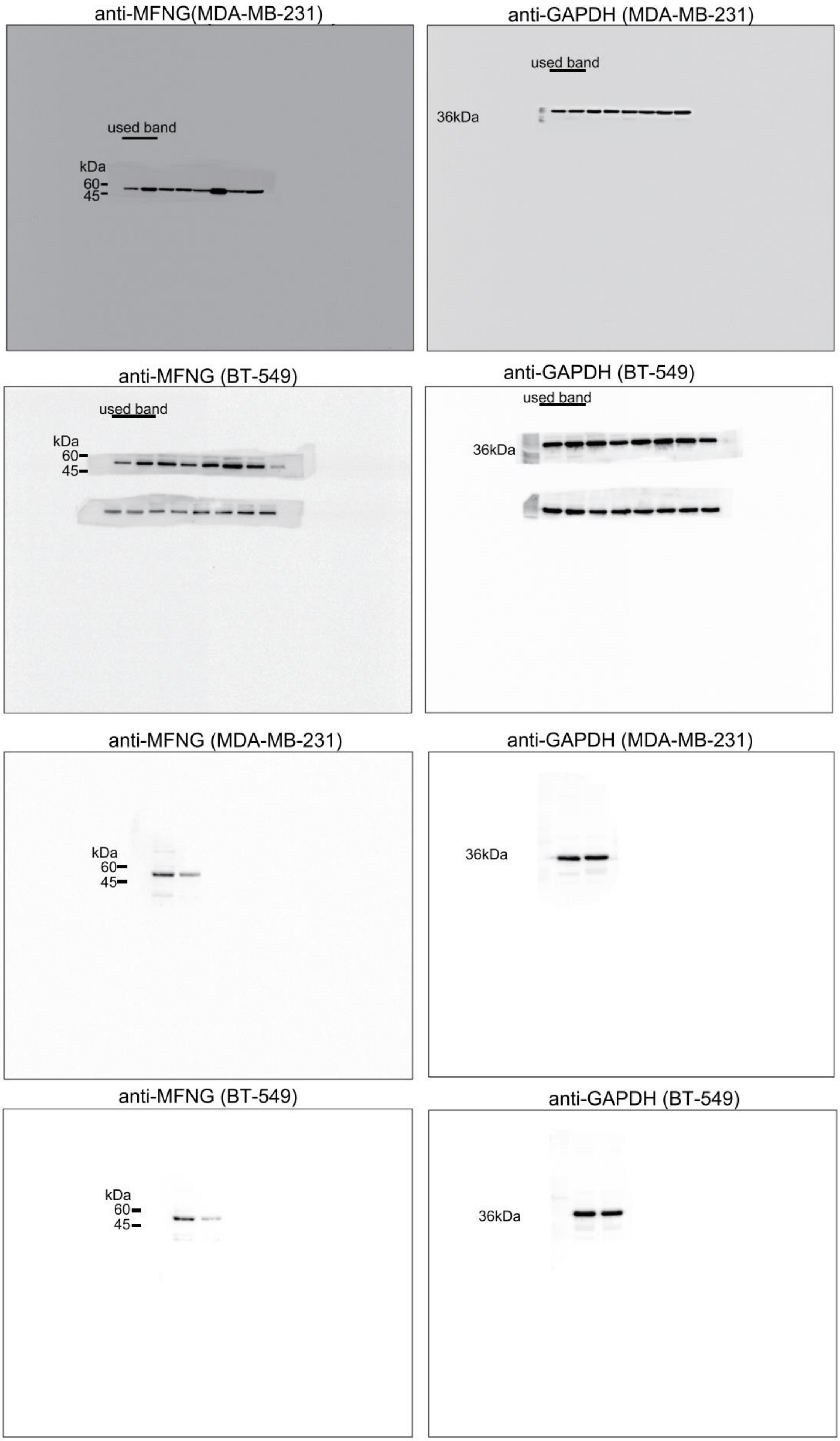

anti-MFNG(MDA-MB-231)
used band
kDa
60
45
anti-GAPDH (MDA-MB-231)
used band
36kDa
anti-MFNG (BT-549)
used band
kDa
60
45
anti-GAPDH (BT-549)
used band
36kDa
anti-MFNG (MDA-MB-231)
kDa
60
45
anti-GAPDH (MDA-MB-231)
36kDa
anti-MFNG (BT-549)
kDa
60
45
anti-GAPDH (BT-549)
36kDa

figure 2F

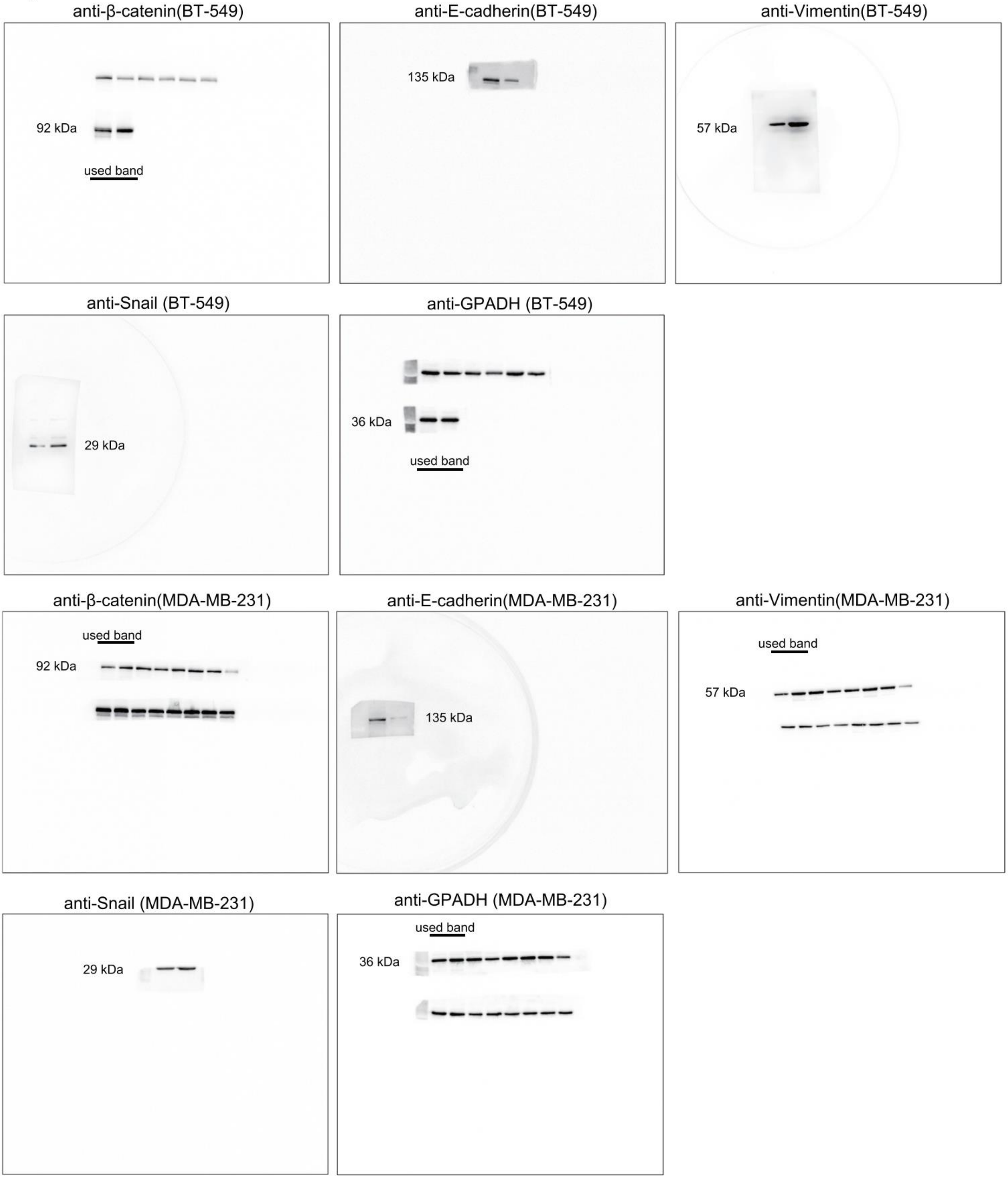
anti-β-catenin(BT-549)
92 kDa
used band
anti-E-cadherin(BT-549)
135 kDa
anti-Vimentin(BT-549)
57 kDa
anti-Snail (BT-549)
29 kDa
anti-GPADH (BT-549)
36 kDa
used band
anti-β-catenin(MDA-MB-231)
used band
92 kDa
anti-E-cadherin(MDA-MB-231)
135 kDa
anti-Vimentin(MDA-MB-231)
used band
57 kDa
anti-Snail (MDA-MB-231)
29 kDa
anti-GPADH (MDA-MB-231)
used band
36 kDa

Figure 3B

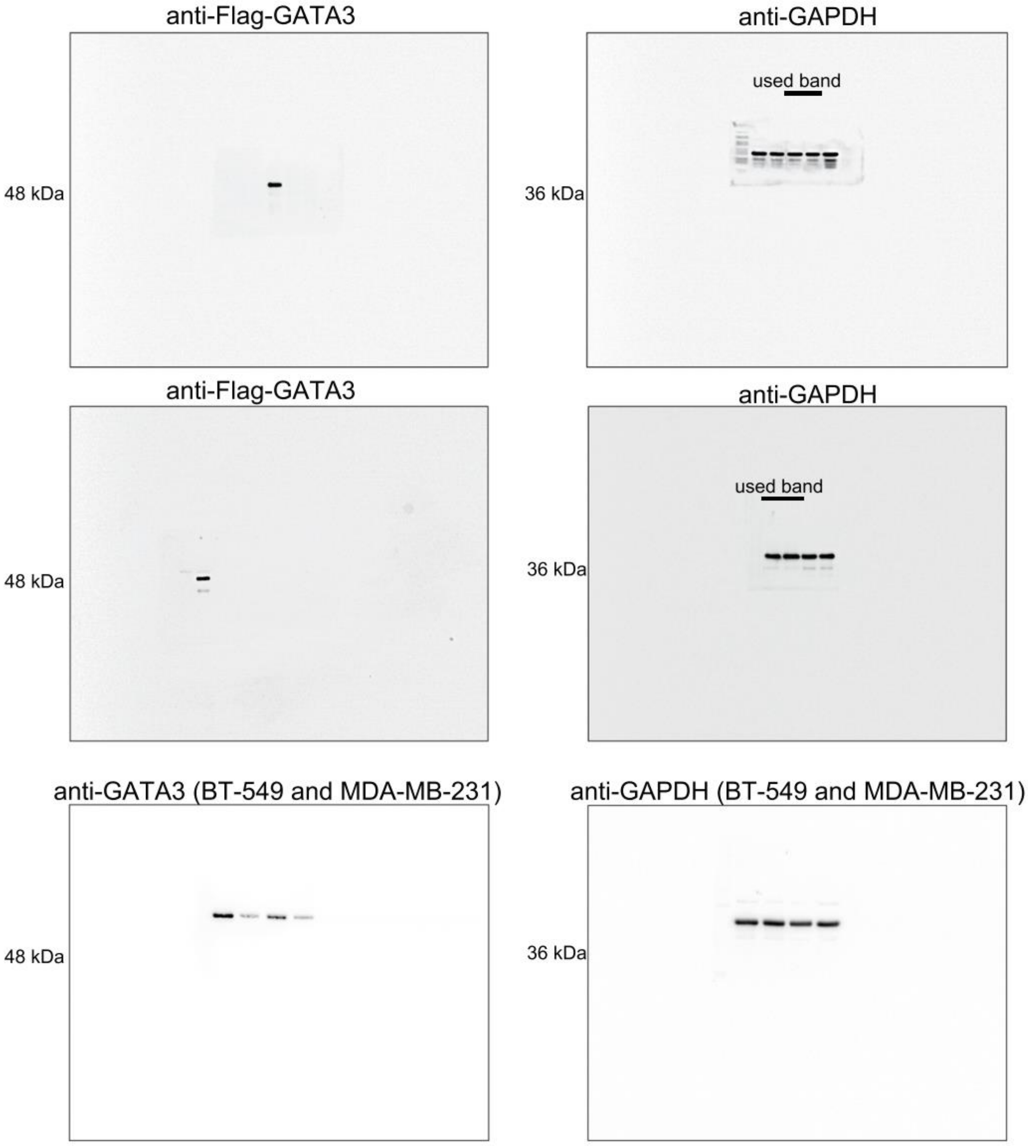
anti-Flag-GATA3
48 kDa
anti-GAPDH
used band
36 kDa
anti-Flag-GATA3
48 kDa
anti-GAPDH
used band
36 kDa
anti-GATA3 (BT-549 and MDA-MB-231)
48 kDa
anti-GAPDH (BT-549 and MDA-MB-231)
36 kDa

Figure 3F

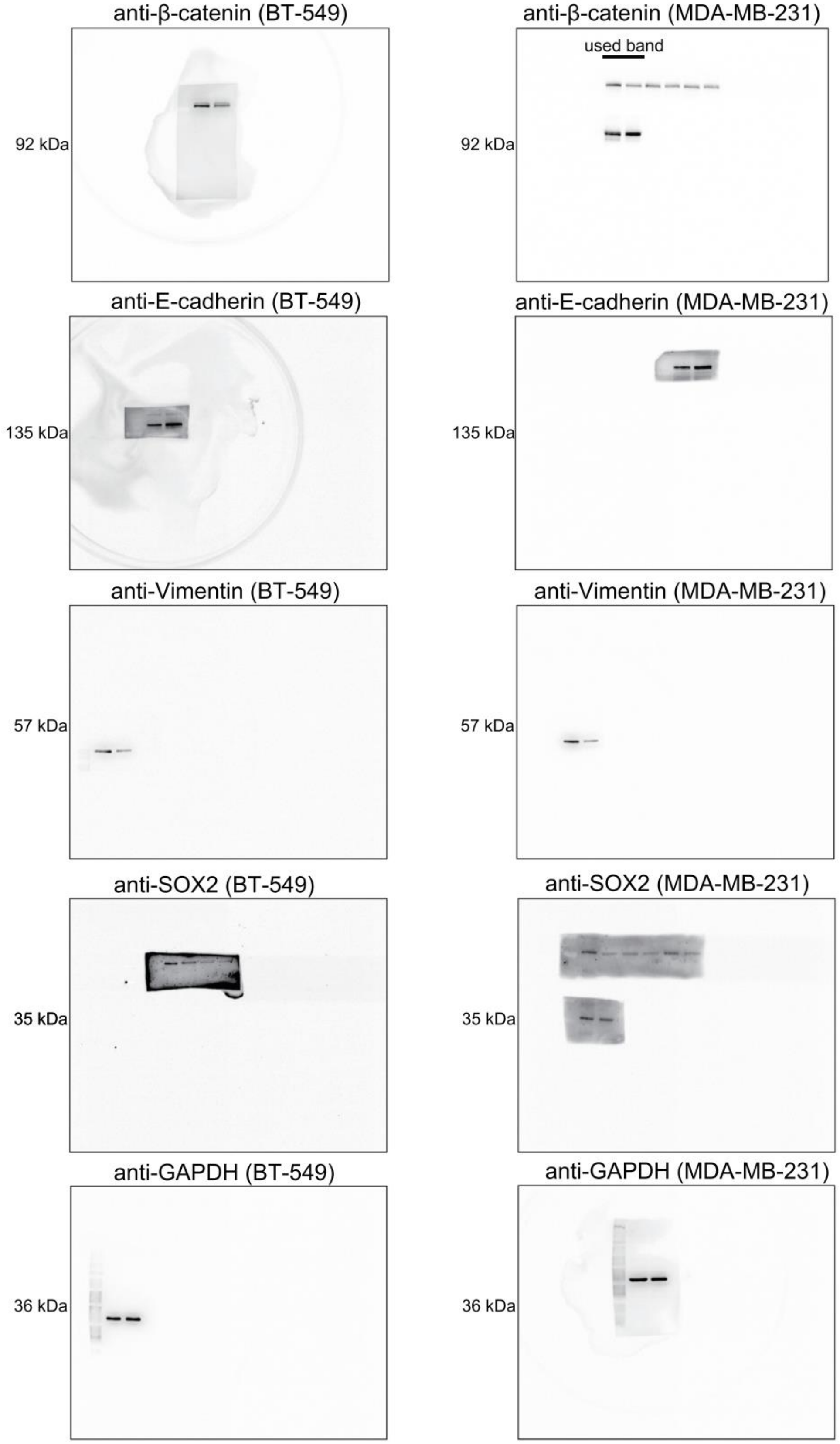
anti-β-catenin (BT-549)
92 kDa
anti-β-catenin (MDA-MB-231)
used band
92 kDa
anti-E-cadherin (BT-549)
135 kDa
anti-E-cadherin (MDA-MB-231)
135 kDa
anti-Vimentin (BT-549)
57 kDa
anti-Vimentin (MDA-MB-231)
57 kDa
anti-SOX2 (BT-549)
35 kDa
anti-SOX2 (MDA-MB-231)
35 kDa
anti-GAPDH (BT-549)
36 kDa
anti-GAPDH (MDA-MB-231)
36 kDa

Figure 4D

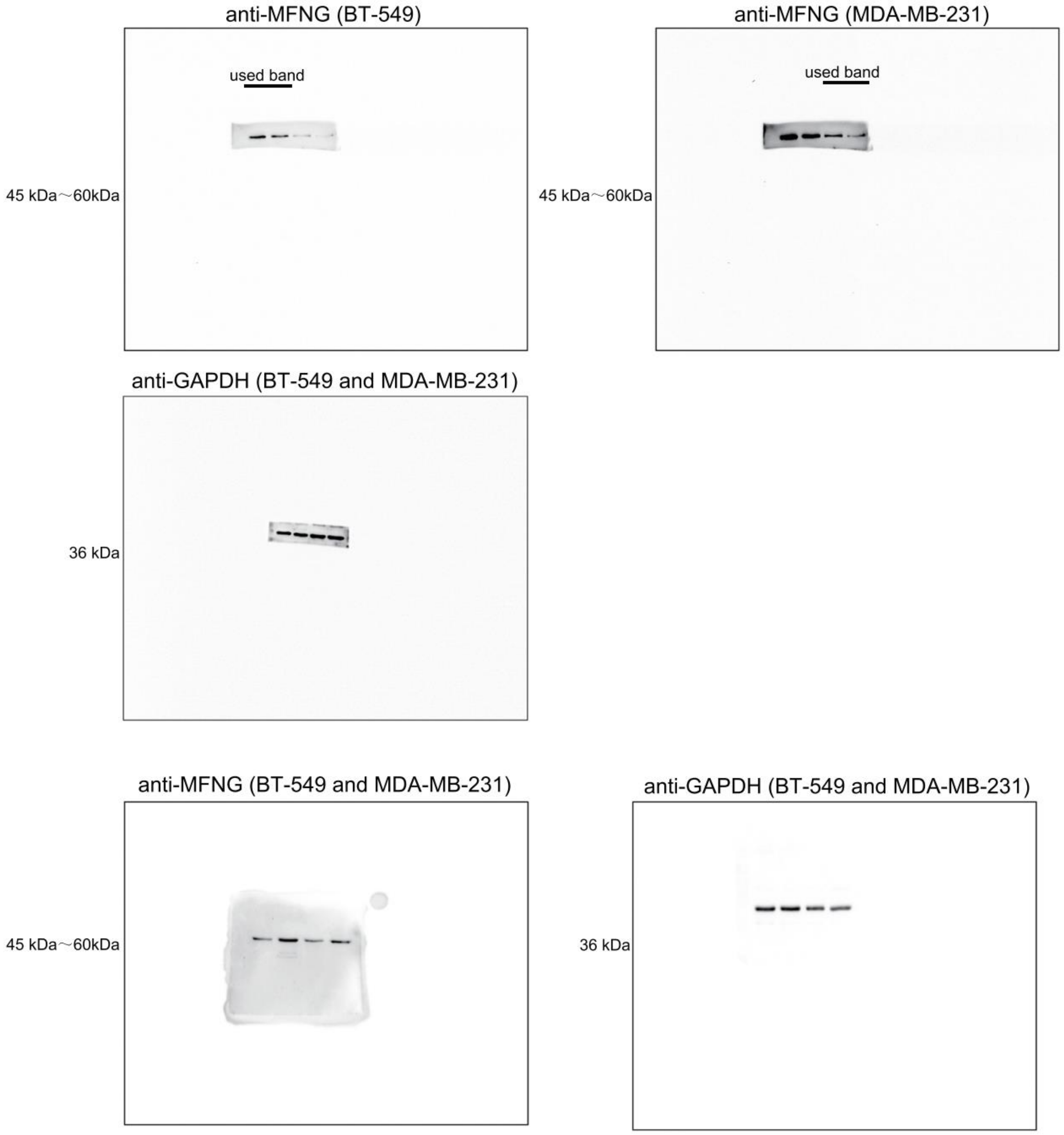
anti-MFNG (BT-549)
used band
45 kDa～60kDa
anti-MFNG (MDA-MB-231)
used band
45 kDa～60kDa
anti-GAPDH (BT-549 and MDA-MB-231)
36 kDa
anti-MFNG (BT-549 and MDA-MB-231)
45 kDa～60kDa
anti-GAPDH (BT-549 and MDA-MB-231)
36 kDa

Figure 5C

anti-GAPDH (BT-549)

36 kDa

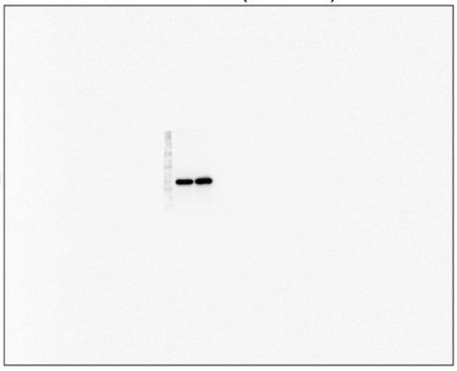

anti-GAPDH (MDA-MB-231)

36 kDa

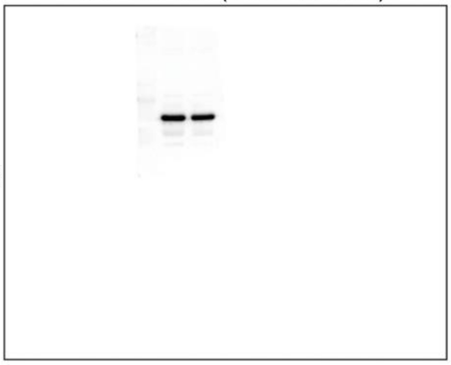

anti-MFNG (BT-549 and MDA-MB-231)

45kDa～60kDa

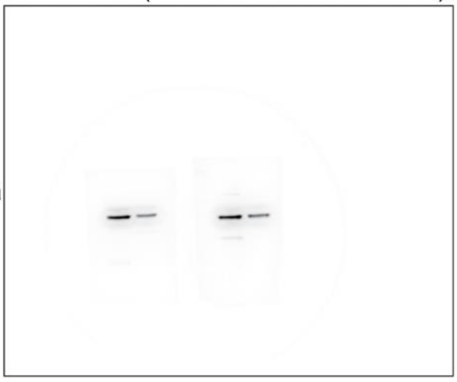

Figure 6E

anti-MFNG (BT-549 and MDA-MB-231)

45kDa～60kDa

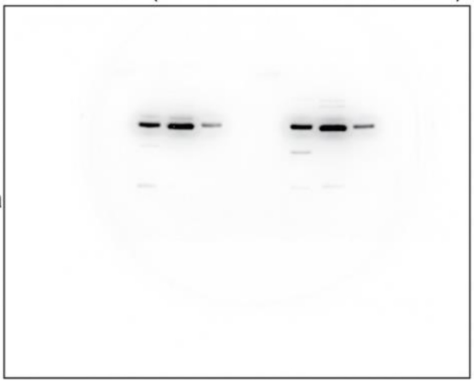

anti-GAPDH (BT-549 and MDA-MB-231)

36 kDa

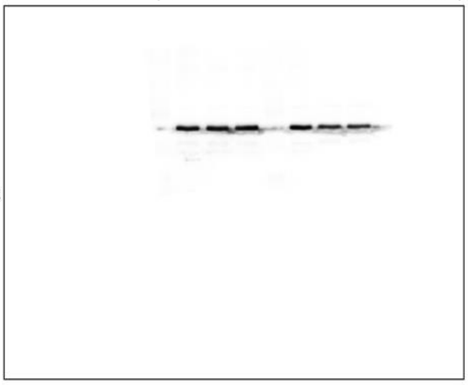

Figure 6H

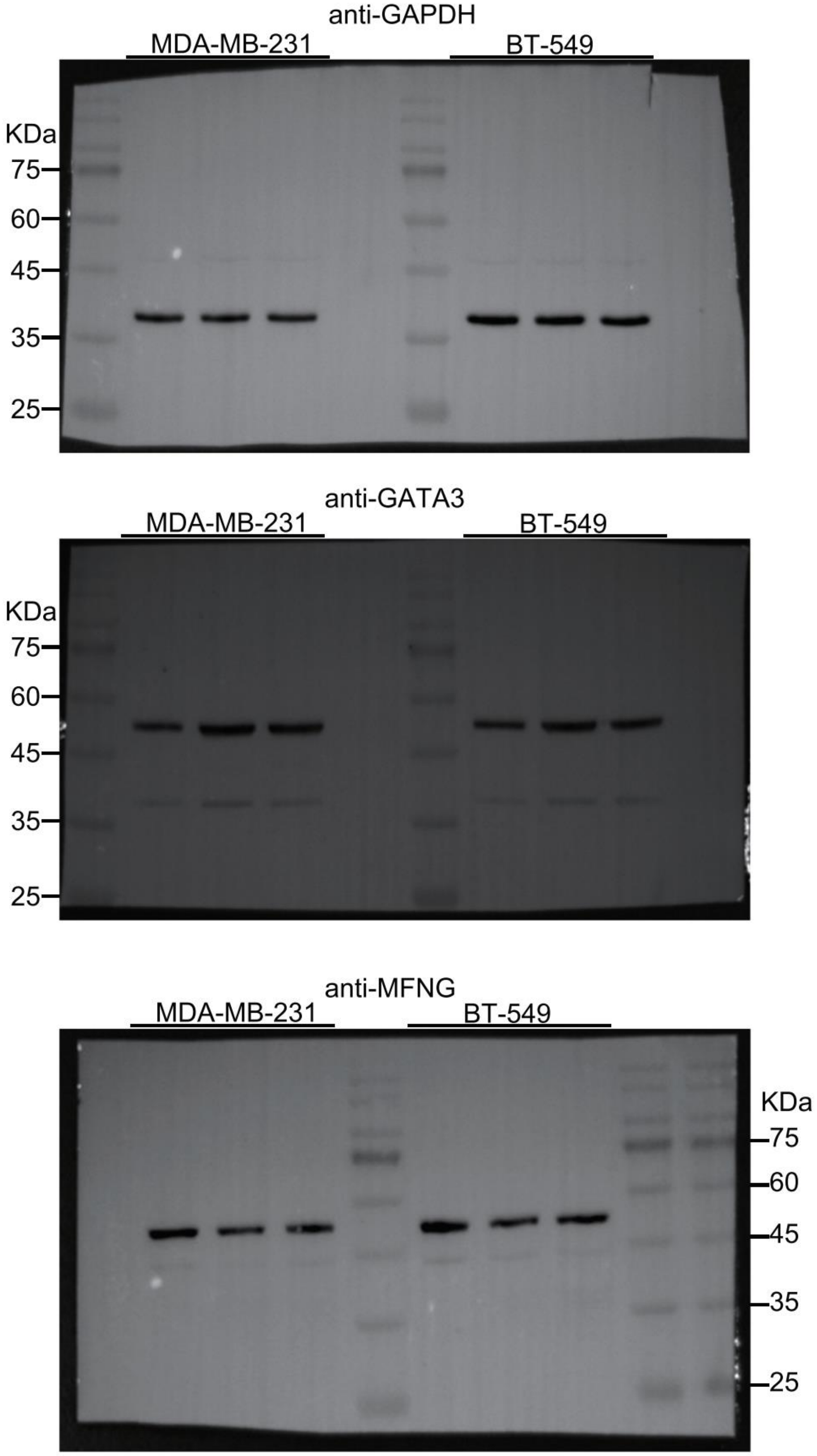
anti-GAPDH
MDA-MB-231
BT-549
KDa
75
60
45
35
25
anti-GATA3
MDA-MB-231
BT-549
KDa
75
60
45
35
25
anti-MFNG
MDA-MB-231
BT-549
KDa
75
60
45
35
25

Figure 7J

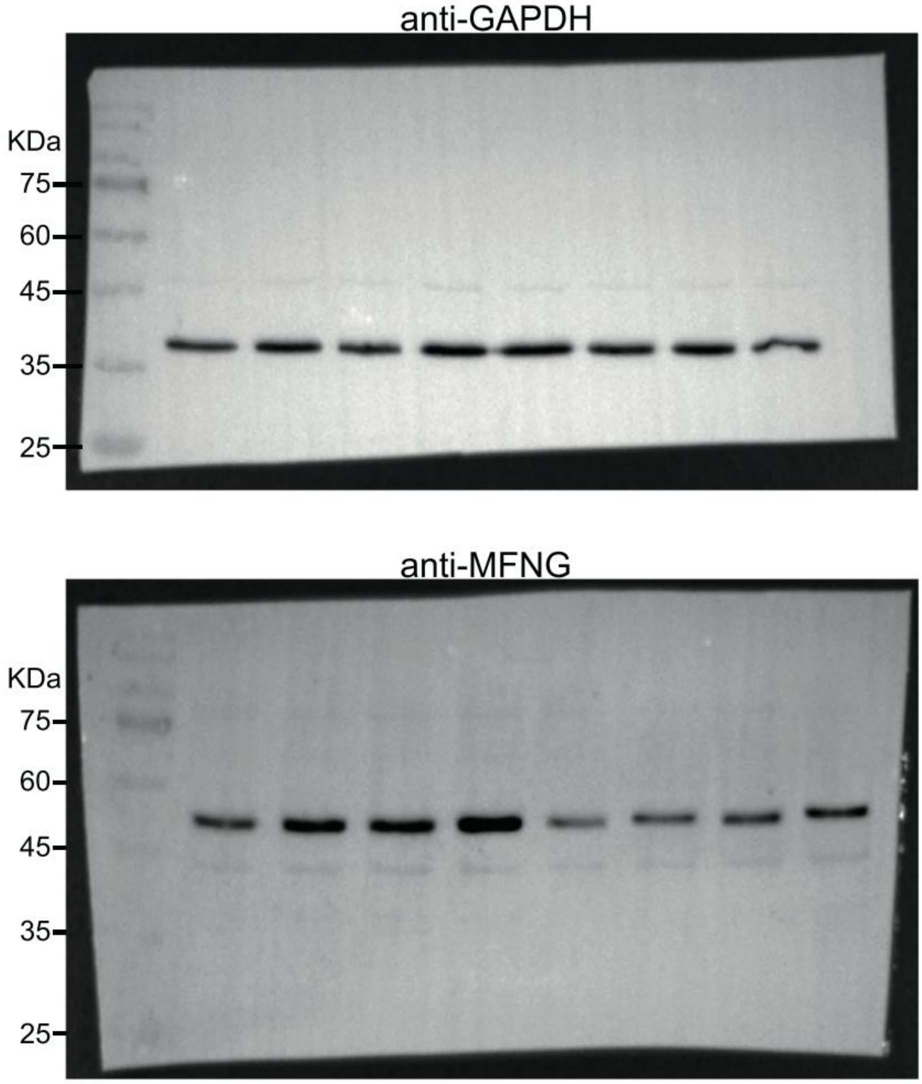

Figure S2. Original uncropped Western blots.

Table S1. Primers for MFNG shRNA and GATA3 shRNA.

Primers	Sequence
Scramble	F: 5′-CCGGCCTAAGGTTAAGTCGCCCTCGCTCGAGC GAGGGCGACTTAACCTTAGG-3′<br>R: 5'-AATTCCCTAAGGTTAAGTCGCCCTCGCTCGAGC GAGGGCGACTTAACCTTAGG-3'
shMFNG	F:5′-CCGGGGGAAACTCAACGTCATTAAGCTCGAGCTTAATGACGTTGAGTTTCCCTTTTTG-3′<br>R: 5'-AATTCAAAAAGGGAAACTCAACGTCATTAAGCTCGAGCTTAATGACGTTGAGTTTCCC-3'
shGATA3	F: 5′-CCGGCCCAAGAACAGCTCGTTTAACCTCGAGGTTAAACGAGCTGTTCTTGGGTTTTTG-3′<br>5'- AATTCAAAAACCCAAGAACAGCTCGTTTAACCTCGAGGTTAAACGAGCTGTTCTTGGG-3'

Table S2. Primers for RT-qPCR; MFNG expression clinical data file (see the single excel file).

Primers	Forward (5′ to 3′)	Reverse (3′ to 5′)
MFNG	TGCTGAGTTCGACACCTTCTT	GCCCTTGGGTTCACATAGTTG
GATA3	GCCCCTCATTAAGCCCAAG	TTGTGGTGGTCTGACAGTTCG

E-cadherin	GACGCCATCAACACCGAGTT	CTTTGTCGTTGGTTAGCTGGT
SLUG	CGAACTGGACACACATACAGTG	CTGAGGATCTCTGGTTGTGGT
TWIST	GTCCGCAGTCTTACGAGGAG	GCTTGAGGGTCTGAATCTTGCT
HES1	TCAACACGACACCGGATAAAC	GCCGCGAGCTATCTTTCTTCA
HEY1	GTTCGGCTCTAGGTTCCATGT	CGTCGGCGCTTCTCAATTATTC
miR205-5p mimic	UCCUUCAUUCCACCGGAGUCUG	CAGACUCCGGUGGAAUGAAGGA
miR-205-5 p inhibitor	CAGACUCCGGUGGAAUGAAGGA	AGGAAGUAAGGUGGCCUCAGAC
SOX2	GCCGAGTGGAAACTTTTGTCG	GGCAGCGTGTACTTATCCTTCT
GAPDH	GGAGCGAGATCCCTCCAAAA	GGCTGTTGTCATACTTCTCATGG
